# Compound Mutation in Cardiac Sarcomere Proteins Is Associated with Increased Risk for Major Arrhythmic Events in Pediatric Onset Hypertrophic Cardiomyopathy

**DOI:** 10.3390/jcm10225256

**Published:** 2021-11-11

**Authors:** Kathrin Pollmann, Emanuel Kaltenecker, Julia Schleihauf, Peter Ewert, Agnes Görlach, Cordula M. Wolf

**Affiliations:** 1 German Heart Center Munich, Department of Congenital Heart Disease and Pediatric Cardiology, School of Medicine & Health, Technical University of Munich, 80636 Munich, Germany; pollmann.k@web.de (K.P.); e.kaltenecker@web.de (E.K.); julia.schleihauf@googlemail.com (J.S.); ewert@dhm.mhn.de (P.E.); goerlach@dhm.mhn.de (A.G.); 2 DZHK (German Centre for Cardiovascular Research), Partner Site Munich Heart Alliance, 80802 Munich, Germany; 3 Experimental and Molecular Pediatric Cardiology, Technical University of Munich, 80636 Munich, Germany

**Keywords:** pediatric onset hypertrophic cardiomyopathy, major arrhythmic events, sudden cardiac death risk stratification, genotype–phenotype association

## Abstract

Hypertrophic cardiomyopathy (HCM) is associated with adverse left ventricular (LV) remodeling causing dysfunction and malignant arrhythmias. Severely affected patients present with disease onset during childhood and sudden cardiac death risk (SCD) stratification is of the highest importance in this cohort. This study aimed to investigate genotype–phenotype association regarding clinical outcome and disease progression in pediatric onset HCM. Medical charts from forty-nine patients with pediatric HCM who had undergone genetic testing were reviewed for retrospective analysis. Demographic, clinical, transthoracic echocardiographic, electrocardiographic, long-term electrocardiogram, cardiopulmonary exercise test, cardiac magnetic resonance, and medication data were recorded. Childhood onset HCM was diagnosed in 29 males and 20 females. Median age at last follow-up was 18.7 years (range 2.6–51.7 years) with a median follow-up time since diagnosis of 8.5 years (range 0.2–38.0 years). Comparison of patients carrying mutations in distinct genes and comparison of genotype-negative with genotype-positive individuals, revealed no differences in functional classification, LV morphology, hypertrophy, systolic and diastolic function, fibrosis and cardiac medication. Patients with compound mutations had a significantly higher risk for major arrhythmic events than a single-mutation carrier. No association between affected genes and disease severity or progression was identified in this cohort.

## 1. Introduction

Hypertrophic cardiomyopathy (HCM) is the most common genetically inherited heart disease with a prevalence of about 0.2% [1,2]. It is defined by isolated hypertrophy and progressive pathologic remodeling of the left ventricular (LV) myocardium [2]. Clinical signs and symptoms include systolic and diastolic ventricular dysfunction and an increased risk for malignant arrhythmias. Disease course is highly variable, and onset usually occurs during adulthood. Severely affected patients present with childhood onset HCM, which is associated with significant lifetime morbidity and mortality.

Disease-causing mutations inherited in an autosomal dominant manner are currently identified in about 60% of HCM patients [2]. They are located predominantly in genes, encoding for essential cardiac sarcomere proteins [1] of cardiomyocytes, most frequently ß-myosin heavy chain (MYH7) and myosin binding protein C (MYBPC3) [3,4]. Other genes such as cardiac troponin T2 (TNNT2) [5], cardiac troponin I3 (TNNI3) [6], cardiac troponin C (TNNC1) [7], myosin light chain 2 (MYL2) [3], and α tropomyosin (TPM1) [3] have also been identified as disease-causing.

The wide range of genes that can be affected by mutations makes HCM a genetically heterogeneous disease [8]. Genetic variability might be a reason for the large spectrum of diverse phenotypes with various clinical outcomes [9]. HCM is known as a disease with variable progression, which can be classified into different stages [8]. Pathological LV remodeling occurs over a lifetime with mostly severe asymmetric septal hypertrophy, contributing to serious left ventricular outflow tract obstruction (LVOTO), myocardial fibrosis, systolic dysfunction with altered LV ejection fraction (EF), diastolic dysfunction including atrial dilatation as well as atrial fibrillations and LV apical aneurysms [8]. Increased morbidity and mortality in the terminal stage of HCM is common due to life-threatening arrhythmias, heart failure, and an increased risk of sudden cardiac death (SCD), especially in youth and competitive athletes [2,10].

Specifically, in pediatric onset HCM, precise knowledge about disease progression, depending on different mutations, is still lacking. Insight of HCM genotype–phenotype association would facilitate counseling and management of affected patients.

This study aimed to investigate genotype–phenotype association of disease severity and progression in patients with childhood onset HCM.

## 2. Materials and Methods

A total of 49 patients diagnosed with pediatric onset HCM between November 1981 and November 2019 at the outpatient clinic of the German Heart Center Munich were included in the study. Pediatric onset HCM was defined as either positive molecular-genetic testing or evidence of disease phenotype before the age of 18 years. Phenotype-positive was defined by the presence of isolated hypertrophied left ventricle with a z-score of ≥2 on transthoracic echocardiographic evaluation based on the guidelines of the European Society of Cardiology (ESC) [11] and guidelines for the diagnosis and treatment of patients with HCM of the American Heart Association and the American College of Cardiology [12]. Accordingly, patients with secondary factors, leading to equivalent LV hypertrophy and the presence of other complex congenital heart disease, syndromic, metabolic, or neuromuscular disorders were excluded.

Data collection was performed retrospectively by medical chart review including demographic and clinical status, transthoracic echocardiography (TTE), electrocardiogram (ECG), 24-h Holter ECG, cardiopulmonary exercise testing (CPET), and cardiac magnetic resonance imaging (CMR). To assess disease progression, patient-related information was obtained at first presentation at the outpatient clinic and compared to information available from last follow-up in those patients, where information for both timepoints was available. CMR data were obtained from 31 patients (63.3%). Since CMR was performed at only one time point, data could not be used for the analysis of disease progression.

**Figure 1:**
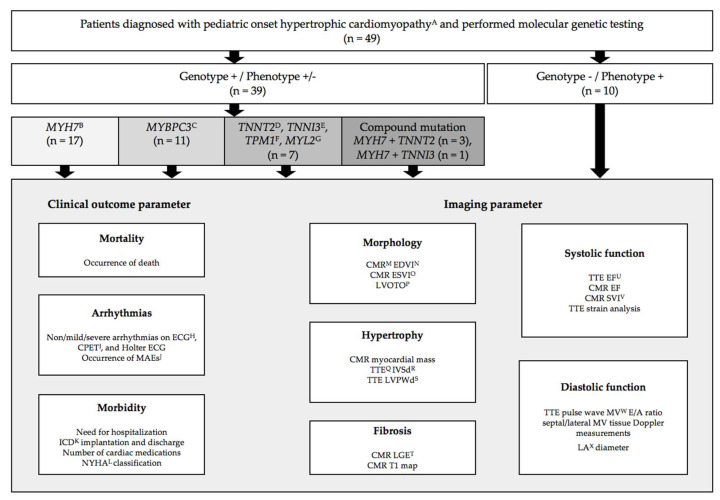
Study design and population with disease-causing mutations in affected genes encoding for sarcomere proteins in the myocardium. Shown are the flow chart of study design and population as well as the distribution of disease-causing mutations in genes encoding for sarcomere proteins in the myocardium as schematic illustrations of the affected proteins of the contractile apparatus of the cardiomyocyte in relation to their location of the thick and thin filament: β myosin heavy chain (MYH7), myosin binding protein C (MYBPC3), cardiac troponin T2 (TNNT2), cardiac troponin I3 (TNNI3), α tropomyosin (TPM1), myosin light chain 2 (MYL2). ^A^, isolated hypertrophic cardiomyopathy according to current European Society of Cardiology and American Heart Association definitions; ^B^, β myosin heavy chain; ^C^, myosin binding protein C; ^D^, cardiac troponin T2; ^E^, cardiac troponin I3; ^F^, α tropomyosin; ^G^, myosin light chain 2; ^H^, electrocardiogram; ^I^, cardiopulmonary exercise test; ^J^, major arrhythmic events: reanimation and/or appropriate discharge of cardioverter-defibrillator and/or sudden cardiac death; ^K^, implantable cardioverter defibrillator; ^L^, New York Heart Association; ^M^, cardiac magnetic resonance imaging; ^N^, end-diastolic volume index; ^O^, end-systolic volume index; ^P^, left ventricular outflow tract obstruction; ^Q^, transthoracic echocardiography; ^R^, end-diastolic septal wall thickness; ^S^, end-diastolic left ventricular posterior wall thickness; ^T^, late gadolinium enhancement; ^U^, ejection fraction; ^V^, stroke volume index; ^W^, mitral valve; ^X^, left atrial.

Clinical and imaging parameters available to assess the presence of pathological myocardial remodeling included end-diastolic and end-systolic LV diameters on CMR and TTE for general cardiac morphology, CMR mass (g/m²) and TTE end-diastolic LV wall thicknesses (z-score) for myocardial hypertrophy, CMR late gadolinium enhancement (LGE) for patchy and CMR T1 map for interstitial myocardial fibrosis, echocardiographic pulse wave mitral valve (MV) E/A ratio and septal/lateral MV tissue Doppler measurements for LV diastolic function, TTE EF and strain analysis for LV systolic function, presence of arrhythmias on ECG, CPET, and 24-h Holter ECG, and clinical functional status assessed by the New York Heart Association (NYHA) class or modified age-adjusted Ross classification [13], medication use, implantable cardioverter defibrillator (ICD) implantation and appropriate discharge, and the need to hospitalization to assess morbidity (Figure 1) [8]. Arrhythmia on Holter or cardiopulmonary exercise test was classified into none, mild (premature ventricular or supraventricular beats), and severe (non-sustained or sustained ventricular or supraventricular tachycardia). Major arrhythmic events (MAEs) were defined as at least one reanimation or appropriate ICD discharge or SCD, in which an appropriate ICD discharge was for ventricular fibrillation or ventricular tachycardia. According to the guidelines of the American College of Cardiology Foundation and the American Heart Association, LVOTO was defined [14].

**Figure 2:**
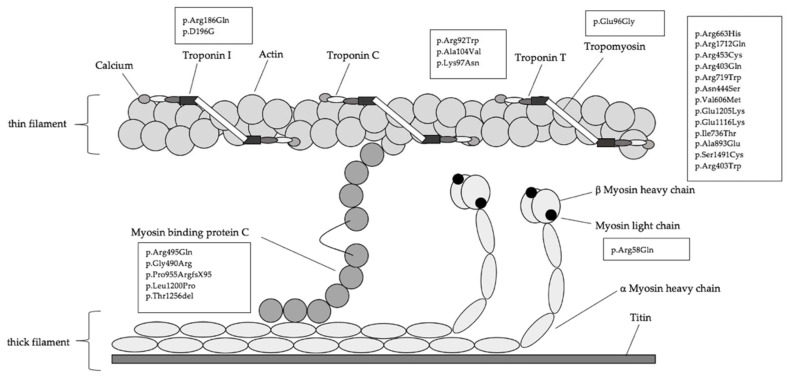
Specific protein modifications of the study population.

Routine molecular genetic testing was performed for the identification of causative genes [4,15]. One mL of the EDTA blood samples were obtained from all patients for genetic testing at certified laboratories for human genetics, following the recommendations of the European and North American guidelines [11,16,17,18]. Interpretation of mutation pathogenicity was based on the classification system and guidelines of the American College of Medical Genetics (ACMG) and Genomics and the Association for Molecular Pathology (AMP) [19,20]. Patients were classified into subgroups based on the gene affected on genetic testing (Figure 1 and Figure 2). Please see the Supplementary Materials for further information (Methods S1).

Statistical analysis was performed with the SPSS software program version 25.0.0 (SPSS Inc., IBM Company, Chicago, IL, USA). Differences between all groups defined at latest follow-up were analyzed by the Kruskal–Wallis test. For comparison of two individual selected groups, the Mann–Whitney U test was utilized. Categorical variables were analyzed by Pearson Chi-square test. With the help of the Kaplan–Meier calculation, the survival probability was estimated and compared between the groups by log-rank test. Continuous variables were expressed as median (minimum–maximum). For assessment of disease progression, the delta of respective parameters between the date of first presentation and last follow-up was compared between groups using the dependent non-parametric Kruskal–Wallis test. Statistical tests were two sided and a *p*-value of <0.05 was considered statistically significant.

## 3. Results

### 3.1. Patients Characteristics

The study population consisted of 29 male (59.2%) and 20 female (40.8%) patients with a diagnosis of pediatric onset HCM based on either clinical findings and/or positive molecular genetic testing. Genetic testing identified 35 consecutive patients as genotype-positive/phenotype-positive (71.4%), four patients as genotype-positive/phenotype-negative (8.2%), and 10 patients as genotype-negative/phenotype-positive (20.4%) at first presentation. Distribution of the detected mutations are depicted in Figure 1 and Figure 2. In 35 pediatric HCM patients, the identified variant was inherited from one parent (89.7%) and four patients had de novo mutation (10.3%) (Table S1). Additional variants of unknown significance (VUS) and benign variations were present in four patients. Of these, singular VUS was detected in two patients, so they were classified as genotype-negative/phenotype positive. One patient carried one VUS in addition to a MYH7 mutation and one patient had one VUS with two further mutations, both defined as genotype positive. Upon closer verification by ClinVar, hosted by the National Center for Biotechnology Information (NCBI) and founded by the National Institutes of Health (NIH), we identified that most mutations were missense variants, followed by frameshift, deletion, and splice mutations (Table S1) [21]. According to the classification systems, all identified mutations were classified as likely pathogenic and/or pathogenic (Table S1).

Patient characteristics are depicted in Table 1. The median age of the entire cohort at the time of diagnosis was 7.0 years (range 0.0–18.95 years) and at last follow-up 18.7 years (range 2.6–51.7 years). No difference was found in disease onset between pediatric HCM patients carrying mutations in thin filament genes compared to patients with mutations in thick filament genes (*p*-value 0.982). Family history was negative in 15 patients (30.6%), positive for the presence of HCM in 23 patients (46.9%) and for SCD in 11 patients (22.4%) (Table 1).

### 3.2. Genotype–Phenotype Relation of Clinical Outcome and Imaging Parameters

**Figure 3:**
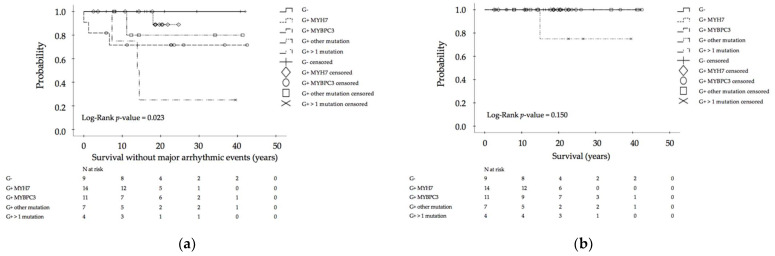
Morbidity and clinical outcome Part I: (**a**) Kaplan–Meier calculation of survival without major arrhythmic events (MAEs) (reanimation or appropriate implantable cardioverter defibrillator discharge or sudden cardiac death) in hypertrophic cardiomyopathy (HCM) patients, depending on different mutations; (**b**) Kaplan–Meier calculation of overall survival; SCD: sudden cardiac death, G−: genotype-negative patients, G+ MYH7: genotype-positive patients with β-myosin heavy chain single-mutation, G+ MYBPC3: genotype-positive patients with myosin binding protein C single-mutation, G+ others: genotype-positive patients with cardiac troponin T2, cardiac troponin I3, α tropomyosin and myosin light chain 2 single-mutations, G+ multiple mutation: genotype-positive patients with compound mutations.

**Figure 4:**
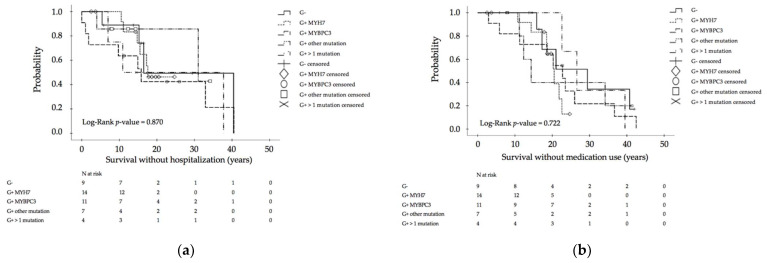
Morbidity and clinical outcome Part II: (**a**) Kaplan–Meier calculation of survival without hospitalization in hypertrophic cardiomyopathy (HCM) patients, depending on different mutations; (**b**) Kaplan–Meier calculation of survival without medication use. SCD: sudden cardiac death, G−: genotype-negative patients, G+ MYH7: genotype-positive patients with β-myosin heavy chain single-mutation, G+ MYBPC3: genotype-positive patients with myosin binding protein C single-mutation, G+ others: genotype-positive patients with cardiac troponin T2, cardiac troponin I3, α tropomyosin and myosin light chain 2 single-mutations, G+ multiple mutation: genotype-positive patients with compound mutations.

Age at first diagnosis and at last follow-up was not different between the distinct patient groups, separated by the respective results of moleculargenetic diagnosis (Table 1). Mortality in general was low (2.0%). One patient carrying a compound mutation of MYH7 and TNNT2 died suddenly at the age of 15 years. Male gender was less present in genotypes other than MYH7 and MYBPC3. A negative family history occurred most often in genotype-negative/phenotype-positive children, a positive family history of HCM more often in children affected by mutations in the MYH7 gene, and a positive family history of SCD more often in patients carrying more than one mutation. Patients experiencing MAEs occurred significantly more often in multiple-mutation carriers than in genotype-negative patients or single-mutation carriers, regardless of mutation type (Table 1, Figure 3). Between defined groups, significant differences with respect to overall survival, survival without hospitalization, and survival without medication use could not be detected (Figure 3 and Figure 4).

There was no difference in the need for hospitalizations and surgery, medication use was similar between groups, and the majority of patients were in NYHA/Ross class I or II at latest follow-up without a difference between groups. Additionally, no differences were found with regard to LV morphology (end-diastolic and end-systolic diameters or presence of LVOTO), myocardial hypertrophy and fibrosis, LV systolic and diastolic function parameters, and the occurrence of arrhythmias on ECG, 24-h Holter ECG or CPET, when comparing patients grouped according to the affected gene (Table 1 and Table 2).

To evaluate gender-difference in disease presentation of pediatric onset HCM patients, a comparison of female (*n* = 20) and male (*n* = 29) patients was performed. Analysis of clinical outcome and imaging parameter did not reach significance, which might be due to the small sample size. Furthermore, investigation of whether there are sex-differences between females and males carrying mutations in the same gene did not yield significant results.

Since carriers with compound mutations were identified at increased risk for MAEs, a subgroup analysis was carried out comparing clinical and imaging findings between patients with one compared to patients with more than one pathogenic/likely pathogenic mutation. Patients with compound mutations had increased maximal wall thickness with higher end-diastolic interventricular septal z-scores at latest follow-up compared to single-mutation carrier (*p*-value 0.05). Additionally, there was a difference in LGE localization measured by CMR (*p*-value 0.019). Patients with single mutations were LGE-positive mainly in the septal area, whereas LGE was positive in the entire myocardium in compound-mutation carrier. There was a trend toward increased left atrial parameters and decreased EF in TTE in patients with compound mutations compared to single-mutation carriers, but the findings did not reach statistical significance, possibly due to small patient numbers (*p*-value 0.056). Detailed descriptive information regarding clinical characteristics, outcome, and imaging parameter of patients with compound mutation are displayed in Table S2.

All multiple-mutation carriers comprised one mutation in the troponin gene in addition to a MYH7 mutation (Figure 1). To assess the contributing influence of a troponin mutation, an additional analysis was performed comparing patients with single or compound mutation in at least one gene encoding a cardiac troponin with patients not carrying a troponin mutation. Patients with a troponin mutation had a positive family history for SCD under the age of 40 more often than patients carrying mutations in other cardiac sarcomere proteins (*p*-value 0.010). Less patients with troponin mutation displayed LVOTO compared to patients with non-troponin mutations (*p*-value 0.032).

In order to differentiate between the effect of a single troponin mutation compared to a troponin mutation in addition to a second mutation, we compared the patients with a single troponin mutation with those carrying multiple mutations. Only patients with compound mutations including a troponin mutation and no patient with a single troponin mutation experienced MAEs (*p*-value 0.018).

### 3.3. Genotype–Phenotype Assessment for Disease Progression

**Figure 5:**
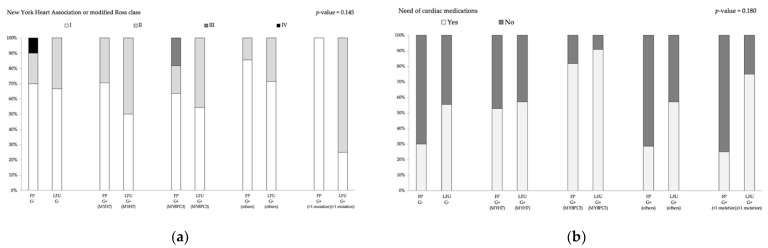

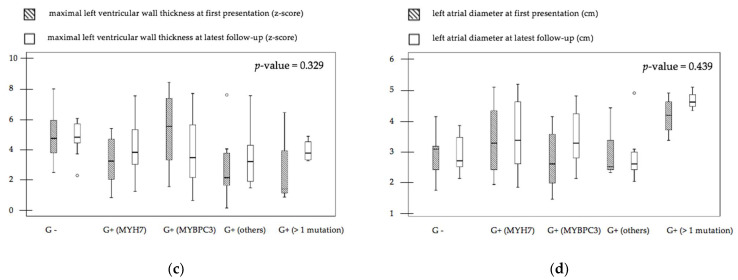
Disease progression Part I. Clinical outcome parameter of pediatric onset HCM patients are presented by the (**a**) New York Heart Association or modified Ross class and by (**b**) the need of cardiac medications. Imaging parameters are presented by (**c**) maximum myocardial wall thickness and by (**d**) the left atrial diameter measured by transthoracic echocardiography. *p*-values were calculated with the non-parametric Kruskal–Wallis test. The delta of the respective parameter between first presentation (FP) and last follow up (LFU) did not differ between consecutive groups. G−: genotype-negative patients, G+ MYH7: genotype-positive patients with β-myosin heavy chain single-mutation, G+ MYBPC3: genotype-positive patients with myosin binding protein C single-mutation, G+ others: genotype-positive patients with cardiac troponin T2, cardiac troponin I3, α tropomyosin and myosin light chain 2 single-mutations, G+ multiple mutation: genotype-positive patients with compound mutations, °: outliers.

After examination of individual groups carrying diverse mutations, indicating no genotype–phenotype association, we additionally examined whether disease progression (i.e., myocardial hypertrophy, fibrosis, systolic and diastolic dysfunction, or presence of arrhythmias) differed depending on mutation type and count. For this, differences between clinical and imaging parameters at follow-up and first presentation were calculated in those patients, where data were present for both timepoints. Based on the results of the statistical analysis, no evidence of statistically significant differences in disease progression of different patient groups could be found (Figure 5, Figures S1 and S2).

## 4. Discussion

The purpose of this study was to investigate genotype–phenotype association in patients with childhood onset HCM, depending on mutation type and count. Furthermore, we examined disease progression in these individuals, especially the dependency of different mutations on disease severity. Genotype–phenotype relationship and predictors for disease progression in adult patients have been previously described, but precise data of children with HCM are rare. This study adds to the body of evidence about genotype–phenotype association in patients diagnosed with HCM during childhood.

The current study revealed no differences with regard to clinical disease course and myocardial pathology comparing patients carrying a single mutation in distinct genes. These findings are in line with other studies mostly including adult HCM patients stating that mutation type is negligible for clinical phenotype and prognosis prediction [22,23,24]. Van Driest et al. categorized genotype-positive tested patients in myofilament-based subgroups for comparison [25]. Concomitant to the results of the current study, researchers faced difficulties to detect clear differences in clinical phenotype. Similar findings of phenotypic independency and tremendous genetic heterogeneity were also determined by comparing MYH7 with MYBPC3 mutation carrier in a study cohort of adults with HCM [26]. In contrast to those findings, authors of a pediatric HCM investigation suggest that pathogenic variants in the MYH7 gene might have a greater impact on phenotypic severity and worse clinical outcome [27]. Earlier disease onset and greater severity of HCM phenotype was identified more frequently in patients with MYH7 variants [27]. One reason for the absence of genotype–phenotype association in our study could be the small sample size of pediatric HCM patients. However, HCM is, in general, caused by rare mutations and the prevalence of variability of mutations in particular gene domains is low, which remains the challenge of detecting clear genotype–phenotype correlation [18]. Taken together, no clear genotype–phenotype relationship in pediatric HCM patients has been established based on current data. More so, data suggest that multiple other non-genetic factors and gene modifications are playing a pivotal role in phenotypic expression [22].

A major finding of the current study is that pediatric patients carrying more than one disease-causing mutation, one of which affects the troponin proteins, are at substantially increased risk for experiencing MAEs. This finding is in line with a large body of evidence derived mostly from adult studies, where HCM patients carrying compound mutations or mutations in the cardiac troponin T are expected to be at higher risk for life-threating arrythmias and SCD [28,29,30,31,32]. One study including adult and adolescent HCM patients reported that multiple-mutation carriers suffered more than twice as many SCDs as single-mutation carriers [28]. A pediatric study including 16 children with multiple variants also reported a higher rate of major arrhythmic cardiac events when compared to single-variant carriers [27]. In summary, data from the literature and the current study results are consistent, supporting the presumption that multiple variants are worse and affect phenotypic severity more strongly in both the adult as well as childhood onset HCM patients. This further strengthens the theory of a “gene dose effect” even in pediatric HCM. Currently, family screening for first-degree relatives of affected patients with HCM is recommended at the age of 12. The results of the current study, together with the fact that early onset HCM was described in children experiencing MAEs [33], underline the necessity of early clinical and genetic screening in young first-degree relatives to initiate risk stratification, preventing SCD in this high risk pediatric HCM cohort [34].

Patients carrying single-gene mutations encoding troponins in the absence of a second mutation did not display a higher risk for SCD and MAEs in the current study. In addition, troponin-mutation carriers were not affected by a worse myocardial phenotype or more severe disease progression compared to patients with other mutations. One reason for this could be the dilemma with a limited number of troponin T mutation carriers, similar to other studies [35], given the fact that troponin mutations are among the rather rare mutations in HCM [25]. The majority of investigations focused on HCM patients diagnosed in adulthood, complicating generalization and exploration to children with HCM. Nevertheless, the initial studies also described no malignant effect on phenotypes of troponin-mutation carriers and clinical phenotypic variability, supporting our findings [36,37]. Taken together, there is still insufficient data concerning a direct association with a worse phenotype in pediatric HCM patients carrying troponin mutations.

The cause of potentially lethal arrhythmic events such as sustained and non-sustained ventricular tachycardia is still unclear [38]. Adverse LV remodeling including myocardial fibrosis [39,40], cardiomyocyte disarray [35], myocyte automaticity [41], and increased calcium sensitivity [42] are suspected to be key factors. Additionally, rapid progression of childhood onset HCM associated with early adverse cardiac events and invasive interventions are already known [33]. We therefore also investigated whether disease progression was different in the respective groups carrying mutations affecting distinct genes. There was no difference in disease progression when comparing the study patients by their mutations. Even subgroup analysis could not reveal that certain mutations influence the course of the disease differently or worse than other mutations. Interestingly, we did not find a worse disease severity over the follow-up period neither in multiple-mutation carriers nor in patients with troponin mutation. As already known, HCM patients show variable clinical progression. Thereby, several patients remain asymptomatic or with mild symptoms over their lifetimes while others face extensive cardiac hypertrophy and further burdens, along with HCM typical anatomic features as well as remodeling processes [8,27]. Among the other components (e.g., further genetic, epigenetic, and environmental factors) influencing substantial variation, this contributes to clinically heterogeneous presentation and disease-related variability in its individual course, being an explanation for our findings. Taken together, the results of the current study could not show a detectable impact of specific genotypes on the severity of disease progression.

The major limitation of the present study is the small number of studied individuals. A general low prevalence of patients with pediatric onset HCM makes it difficult to obtain a large study cohort and to achieve an adequate informative value. A single-center design was chosen given the heterogeneity in imaging modalities, inter-observer variability in imaging data analysis, and the lack of moleculargenetic diagnosis in a multicenter setting. Additionally, the limitations of a retrospective study in general apply. Comparison to other studies was hampered by the fact that most studies investigating genotype–phenotype relationship in HCM to date have been conducted in adult patients who were not diagnosed with HCM until the age of 18. This affects the comparability of individual study results between childhood and adulthood onset of HCM. Finally, the study results cannot simply be transferred to other centers as this was a single-center study at a tertiary care university hospital.

Despite the limitations, the current study enhances a better understanding of the multifactorial nature of HCM in pediatric patients. Clinical phenotypes of patients with childhood onset HCM is heterogenous and mainly independent of distinct single mutations. As mutation-specific risk stratification still remains a challenge, further research is needed to identify predictors for severe arrhythmias, causing SCD in pediatric patients with HCM.

## 5. Conclusions

In this single center observational study, pediatric patients carrying compound mutations were at increased risk of suffering from MAEs, which drastically increased morbidity and mortality. Molecular genetic testing during childhood can identify those high-risk children and allows for early initiation of preventive measures to avoid the occurrence of life-threatening arrhythmias and SCD.

## Figures and Tables

**Table 1:** Patient characteristics and clinical outcome parameters.

Patients Characteristics and Clinical Outcome Parameters	Genotype- Positive (MYH7)	Genotype- Positive (MYBPC3)	Genotype- Positive (Others)	Genotype- Positive (>1 Mutation)	Genotype-Negative/Phenotype-Positive	*p*-Value
Patients, *n* ^1^ (% of total)	17 (34.7)	11 (22.4)	7 (14.3)	4 (8.2)	10 (20.4)	
Male, *n* (%)	9 (52.9)	10 (90.9)	1 (14.3)	3 (75.0)	6 (60.0)	0.025 ^10^
Age at first diagnosis (years)	13.0 (0.0–19.0)	9.0 (0.0–18.0)	6.8 (0.0–15.0)	3.5 (0.0–7.0)	8.3 (0.0–15.6)	0.674 ^11^
Age at last follow-up (years)	18.7 (2.6–51.7)	22.7 (3.0–42.5)	12.4 (7.9–41.4)	24.6 (14.6–39.6)	18.0 (5.9–42.0)	0.521 ^11^
Follow-up time (years)	5.8 (0.2–22.6)	8.8 (2.3–31.7)	10.3 (1.2–27.3)	21.1 (13.5–33.5)	7.0 (0.2–38.0)	0.188 ^11^
**Family History**						
Negative, *n* (%)	3/17 (17.6)	4/11 (22.4)	1/7 (14.3)	0/4 (0.0)	7/10 (70.0)	
HCM ^2^, *n* (%)	12/17 (70.6)	5/11 (45.5)	2/7 (28.6)	1/4 (25.0)	3/10 (30.0)	
SCD ^3^, *n* (%)	2/17 (11.8)	2/11 (18.2)	4/7 (57.1)	3/4 (75.0)	0/10 (0.0)	0.003 ^10^
**Mortality**						
Death, *n* (%)	0/17 (0.0)	0/11 (0.0)	0/7 (0.0)	1/4 (25.0)	0/10 (0.0)	0.022 ^10^
**Arrhythmia ^4^**						
None, *n* (%)	7/14 (50.0)	3/11 (27.3)	4/7 (57.1)	1/4 (25.0)	6/9 (66.7)	
Mild ^5^, *n* (%)	5/14 (35.7)	4/11 (36.4)	1/7 (14.3)	0/4 (0.0)	3/9 (33.3)	
Severe ^6^, *n* (%)	2/14 (14.3)	4/11 (36.4)	2/7 (28.6)	3/4 (75.0)	0/9 (0.0)	
MAEs ^7^, *n* (%)	1/17 (5.9)	3/11 (27.3)	1/7 (14.3)	3/4 (75.0)	0/10 (0.0)	0.006 ^10^
**Morbidity**						
Hospitalization, *n* (%)	6/17 (35.3)	8/11 (72.7)	2/7 (28.6)	3/4 (75.0)	4/10 (40.0)	0.180 ^10^
Age (years), median (range)	6.5 (1.1–17.3)	1.0 (0.0–27.9)	10.5 (4.0–17.0)	10.9 (0.0–31.8)	9.0 (0.0–34.5)	0.722 ^11^
ICD ^8^, *n* (%)	2/17 (11.8)	4/11 (36.4)	2/7 (28.6)	3/4 (75.0)	2/10 (20.0)	0.112 ^10^
Age (years), median (range)	15.2 (14.8–15.5)	23.9 (9.9–40.5)	17.7 (4.3–31.0)	14.3 (10.9–37.8)	28.5 (16.5–40.4)	0.700 ^11^
Primary prevention, *n* (%)	2/2 (100.0)	4/4 (100.0)	2/2 (100.0)	1/3 (33.3)	2/2 (100.0)	0.096 ^10^
Secondary prevention, *n* (%)	0/2 (0.0)	0/4 (0.0)	0/2 (0.0)	2/3 (66.6)	0/2 (0.0)	0.096 ^10^
Appropriate discharge, *n* (%)	1/2 (50.0)	2/4 (50.0)	1/2 (50.0)	2/3 (66.7)	0/2 (0.0)	0.686 ^10^
Number of cardiac medication, *n* (%)	1 (0–2)	1 (0–2)	1 (0–2)	1 (0–2)	1 (0–2)	0.624 ^10^
NYHA ^9^/Ross class						
I, *n* (%)	7/14 (50.0)	6/11 (54.5)	5/7 (71.4)	1/4 (25.0)	6/9 (66.7)	0.582 ^10^
II, *n* (%)	7/14 (50.0)	5/11 (45.5)	2/7 (28.6)	3/4 (75.0)	3/9 (33.3)	
III, *n* (%)	0/14 (0.0)	0/13 (0.0)	0/7 (0.0)	0/4 (0.0)	0/9 (0.0)	
IV, *n* (%)	0/14 (0.0)	0/13 (0.0)	0/7 (0.0)	0/4 (0.0)	0/9 (0.0)	

^1^, number of cases; ^2^, hypertrophic cardiomyopathy; ^3^, sudden cardiac death; ^4^, arrhythmia on Holter or cardiopulmonary exercise test; ^5^, premature ventricular or supraventricular beats; ^6^, non-sustained or sustained ventricular or supraventricular tachycardia; ^7^, resuscitation and/or appropriate implantable cardioverter defibrillator discharge and/or sudden cardiac death; ^8^, implantable cardioverter defibrillator; ^9^, New York Heart Association or Modified Ross classification according to age; ^10^, Pearson-Chi-squared-test; ^11^, Kruskal–Wallis-test.

**Table 2:** Disease phenotype at latest follow-up.

Imaging Parameter	Genotype- Positive (MYH7) *n* = 17	Genotype- Positive (MYBPC3) *n* = 11	Genotype- Positive (Others) *n* = 7	Genotype- Positive (>1 Mutation) *n* = 4	Genotype-Negative/Phenotype-Positive *n* = 10	*p*-Value
**Morphology**						
CMR ^1^ EDVI ^2^ (mL/m^2^), median (range)	62.0 (50.0–89.0)	57.0 (36.0–91.0)	60.0 (53.0–65.0)	63.5 (63.0–64.0)	65.0 (48.0–76.0)	0.949 ^18^
CMR ESVI ^3^ (mL/m^2^), median (range)	19.5 (8.0–27.0)	17.0 (7.0–35.0)	19.0 (11.0–22.0)	16.0 (16.0–16.0)	17.0 (8.0–27.0)	0.978 ^18^
LVOTO ^4^, *n* ^5^ (%)	7/14 (50.0)	3/11 (27.3)	0/7(0.0)	0/4(0.0)	2/9 (22.2)	0.092 ^19^
**Hypertrophy**						
CMR myocardial mass (g/m^2^), median (range)	100.5 (39.0–168.0)	83.0 (39.0–213.0)	58.0 (48.0–95.0)	80.0 (43.0–117.0)	90.0 (56.0–126.0)	0.602 ^18^
TTE ^6^ IVSd ^7^ z-score, median (range)	3.7 (0.6–7.6)	3.4 (0.2–7.8)	2.2 (0.1–7.6)	3.7 (3.1–4.9)	4.8 (2.2–6.1)	0.536 ^18^
TTE LVPWd ^8^ z-score, median (range)	2.5 (0.7–3.8)	1.7 (-1.0–5.1)	2.1 (1.2–5.3)	2.9 (1.7–3.3)	2.6 (1.1–4.5)	0.891 ^18^
**Fibrosis**						
CMR LGE ^9^, *n* (%)	8/10 (80.0)	8/9 (88.9)	3/3 (100.0)	2/2 (100.0)	4/7 (57.1)	0.396 ^19^
CMR LGE localization						0.204 ^19^
Negative, *n* (%)	3/10 (30.0)	3/9 (33.3)	1/3 (33.3)	0/2 (0.0)	3/7 (42.9)	
Uncertain detection, *n* (%)	3/10 (30.0)	0/9 (0.0)	1/3 (33.3)	1/2 (50.0)	1/7 (14.3)	
Septum, *n* (%)	4/10 (40.0)	4/9 (44.4)	1/3 (33.3)	0/2 (0.0)	2/7 (28.6)	
Entire myocardium, *n* (%)	0/10 (0.0)	0/9 (0.0)	0/3 (0.0)	1/2 (50.0)	0/7 (0.0)	
Papillary muscle + RVOT ^10^, *n* (%)	0/10 (0.0)	1/9 (11.1)	0/3 (0.0)	0/2 (0.0)	0/7 (0.0)	
LV ^11^ front wall + septum, *n* (%)	0/10 (0.0)	0/9 (0.0)	0/3 (0.0)	0/2 (0.0)	1/7 (14.3)	
Diffuse distribution, *n* (%)	0/10 (0.0)	1/9 (11.1)	0/3 (0.0)	0/2 (0.0)	0/7 (0.0)	
CMR LGE mean, median (range)	7.3 (0.6–14.0)	20.2 (0.4–23.8)	7.8 (7.8–7.8)	4.8 (4.8–4.8)	1.2 (1.2–1.2)	0.278 ^18^
CMR ECV ^12^ total mean, median (range)	26.8 (25.9–30.6)	28.6 (24.9–34.4)	28.5 (28.5–28.5)	23.1 (23.1–23.1)	24.8 (24.8–24.8)	0.289 ^18^
CMR ECV septal mean, median (range)	31.1 (27.8–34.0)	28.7 (26.9–34.9)	31.3 (31.3–31.3)	22.1 (22.1–22.1)	26.3 (26.3–26.3)	0.926 ^18^
**Systolic function**						
TTE EF ^13^ (%), median (range)	72.0 (44.0–88.0)	73.0 (55.0–95.0)	76.0 (61.0–88.0)	59.0 (41.0–78.0)	83.0 (59.0–89.0)	0.156 ^18^
CMR EF (%), median (range)	72.5 (62.0.–83.0)	73.0 (61.0–80.0)	69.0 (66.0–80.0)	75.0 (75.0–75.0)	73.0 (65.0–88.0)	0.987 ^18^
CMR SVI ^14^ (mL/m^2^), median (range)	42.5 (35.0–67.0)	45.0 (29.0–59.0)	43.0 (41.0–44.0)	47.0 (47.0–47.0)	46.5 (37.0–59.0)	0.918 ^18^
GLS ^15^ average, median (range)	−16.3 (−22.5–−8.8)	−14.7 (−27.8–−7.4)	−10.1 (−24.1–−7.6)	−8.2 (−8.2–−8.2)	−16.25 −22.2–−11.9)	0.535 ^18^
GLS dispersion, median (range)	−14.5 (−36.0–−4.0)	−11.0 (−24.0–−6.0)	−8.0 (−13.0–−5.0)	−4.0 (−18.0–−2.0)	−11.0 (−19.0–−5.0)	0.299 ^18^
GLS minimum, median (range)	−24.0 (−38.0–−8.0)	−15.0 (−24.0− −6.0)	−18.0 (-33.0–−10.0)	−13.0 (−17.0–−9.0)	−20.0 (−33.0–−9.0	0.345^18^
GLS maximum, median (range)	−8.5 (−20.0–7.0)	−7.0 (−17.0–3.0)	−6.0 (−22.0–−4.0)	−7.0 (−13.0–5.0)	−9.0 (−15.0–10.0)	0.576 ^18^
GLS septal basal, median (range)	−9.5 (−26.0–−4.0)	−7.0 (−17.0–3.0)	−11.0 (−22.0–−5.0)	−8.0 (−16.0–−3.0)	−10.0 (−17.0–−5.0)	0.319 ^18^
GLS septal middle, median (range)	−13.0 (−29.0–−4.0)	−10.0 (−27.0–1.0)	−8.0 (−27.0–−4.0)	−7.0 (−17.0–−4.0)	−12.0 (−25.0–−4.0)	0.687 ^18^
GLS septal apex, median (range)	−18.0 (−32.0–−5.0)	−15.0(−39.0–−8.0)	−14.0 (−33.0–−7.0)	−13.0 (−13.0–−9.0)	−16.0 (−33.0–10.0)	0.849 ^18^
GLS lateral basal, median (range)	−20.5 (−38.0–7.0)	−13.0 (−27.0–−5.0)	−18.0 (−33.0–−7.0)	−1.5 (−8.0–5.0)	−14.0 (−27.0–−6.0)	0.304 ^18^
GLS lateral middle, median (range)	−15.0 (−28.0–3.0)	−18.5 (−32.0–1.0)	−9.5 (−21.0–−6.0)	N/A ^20^	−16.0 (−18.0–−12.0)	0.626 ^18^
GLS lateral apex, median (range)	−15.0 (−30.0–−5.0)	−34.0 (−34–−34.0)	−12.0 (−29.0–−10.0)	−8.0 (−8.0–−8.0)	−16.0 (−20.0–−12.0)	0.360 ^18^
**Diastolic function**						
MV ^16^ E/A Ratio, median (range)	1.4 (0.7–2.2)	1.8 (1.4–3.0)	1.7 (1.0–2.5)	2.0 (1.1–2.0)	1.5 (1.0–12.8)	0.420 ^18^
MV E Deceleration (m/s), median (range)	2.0 (1.1–2.5)	2.0 (1.5–2.9)	2.1 (1.7–2.8)	2.2 (2.0–2.6)	2.0 (1.6–2.9)	0.610 ^18^
MV E maximum (m/s), median (range)	0.8 (0.4–1.3)	0.8 (0.7–1.5)	0.7 (0.6–1.1)	0.6 (0.5–0.8)	0.8 (0.6–1.8)	0.504 ^18^
MV E’ septal (m/s), median (range)	9.5 (5.0–13.0)	5.7 (5.0–7.0)	11.0 (3.0–15.0)	5.5 (4.0–7.0)	7.5 (3.0–12.0)	0.176 ^18^
MV E’ lateral (m/s), median (range)	10.0 (5.0–12.0)	9.0 (7.0–11.0)	7.0 (5.0–18.0)	7.0 (5.0–9.0)	11.5 (7.0–14.0)	0.459 ^18^
E/E’ septal, median (range)	7.9 (5.2–21.8)	12.3 (1.7–14.7)	7.5 (6.1–20.0)	10.5 (8.6–12.5)	11.6 (6.9–19.8)	0.507 ^18^
E/E’ lateral, median (range)	8.0 (5.8–11.9)	8.1 (7.0–9.4)	8.1 (−5.1–12.0)	8.3 (6.7–10.0)	7.3 (5.2–14.1)	0.895 ^18^
LA ^17^ Diameter (cm), median (range)	3.3 (1.7–5.2)	3.2 (2.0–4.8)	2.5 (1.9–4.9)	4.6 (4.3–5.1)	2.6 (2.0–3.8)	0.106 ^18^

^1^, Cardiac magnetic resonance imaging; ^2^, end-diastolic volume index; ^3^, end-systolic volume index; ^4^, left ventricular outflow tract obstruction; ^5^, number of cases; ^6^, transthoracic echocardiography; ^7^, end-diastolic inter-ventricular septal; ^8^, end-diastolic left ventricular posterior wall thickness; ^9^, late gadolinium enhancement; ^10^, right ventricular outflow tract; ^11^, left ventricular; ^12^, extracellular volume fraction; ^13^, ejection fraction; ^14^, stroke volume index; ^15^, global longitudinal strain; ^16^, mitral valve; ^17^, left atrium; ^18^, Kruskal Wallis test; ^19^, Pearson Chi-square test; ^20^, N/A: not available.

## Data Availability

Not applicable.

## Supplementary Materials

The following are available online at https://www.mdpi.com/article/10.3390/jcm10225256/s1, Methods S1: Molecular genetic testing, Table S1: Mutation description of molecular genetic testing of genotype-positive patients with hypertrophic cardiomyopathy, Table S2: Clinical characteristics and imaging parameter of pediatric hypertrophic cardiomyopathy patients with compound mutations, Figure S1: Disease progression Part II, Figure S2: Progress of arrhythmia in patients with pediatric onset hypertrophic cardiomyopathy.
