# Supplementary material for: Compound Mutation in Cardiac Sarcomere Proteins Is Associated with Increased Risk for Major Arrhythmic Events in Pediatric Onset Hypertrophic Cardiomyopathy
Source: J Clin Med. 2021 Nov 11;10(22):5256. doi: 10.3390/jcm10225256 (PMC8617951; doi:10.3390/jcm10225256)
Supplement: Supplementary file 1 [file jcm-10-05256-s001.zip › jcm-1443404-supplementary.pdf]

Supplementary Material

Methods S1: Molecular genetic testing.

Genetic testing strategy included amplification with polymerase chain reaction followed by sanger sequencing in 17 patients (34.7%), next generation sequencing as sequencing by synthesis by illumina in 14 patients (28.6%) and whole exome analysis in 5 patients (10.2%). The type of molecular genetic testing was not available in the case of 13 patient (26.5%). Multiple gene diagnostic was performed in 24 patients. Gene panels included at least sequencing of the *MYH7* and *MYBPC3* genes or the combination of MYH7 and TNNT2. The biggest panel consisted of 16 known causative genes like ACTC1, ACTN2, ANKRD1, CSRP3, JPH2, MYBPC3, MYH7, MYL2, MYL3, PLN, PRKAG2, TCAP, TNNC1, TNNI3, TNNT2 and TPM1. Single gene analysis was conducted in a total of 7 patients, with amplification and sequencing of the MYH7 gene in 4 patients and the TNNT2 gene in 3 patients. Based on the classification system and guidelines of the American College of Medical Genetics and Genomics (ACMG) and The Association for Molecular Pathology (AMP) mutations were interpreted [1,2]. Genotype-positive was defined as the result of molecular genetic testing, revealing a pathogenic or likely pathogenic variant as per ACMG criteria or re-analysis in ClinVar. Patients with negative result of molecular genetic testing and patients with variants of uncertain significance were defined as genotype negative. Genotype-positive/phenotype-positive, genotype-positive/phenotype-negative as well as genotype-negative/phenotype-positive individuals were included for comparison. Patients identified as genotype positive and still phenotype negative at the time of the study were also included, as this cohort has a genetic predisposition to develop a phenotype typical of hypertrophic cardiomyopathy and may still become clinically conspicuous during childhood. In addition, there are patients who have detected a disease-causing mutation and yet do not develop a phenotype, presumably due to less harmful mutational variants within the affected gene or influence of other nongenetic or epigenetic factors.

Table S1. Mutation description of molecular genetic testing of genotype-positive patients with hypertrophic cardiomyopathy.

Gene	Reference Sequence Transcript	Nucleotid change	Protein modification	Mutation type	ClinVar Classification	Number of patients carrying mutation	Mutation origin
MYH7 [1]	NM_000257.3	c.1988G>A	p.Arg663His	missense	pathogenic	1	inherited [8]
	NM_000257.2	c.5135G>A	p.Arg1712Gln	missense	likely pathogenic	1	inherited
	NM_000257.2	c.1357C>T	p.Arg453Cys	missense	likely pathogenic to pathogenic	1	inherited
	NM_000257.3	c.1208G>A	p.Arg403Gln	missense	pathogenic	2	de novo [9], inherited
	NM_000257.3	c.2155C>T	p.Arg719Trp	missense	pathogenic	4	inherited
	NM_000257.4	c.1331A>G	p.Asn444Ser	missense	likely pathogenic	1	inherited
	NM_000257.3	c.1816G>A	p.Val606Met	missense	likely pathogenic to pathogenic	2	inherited
	NM_000257.3	c.3613G>A	p.Glu1205Lys	missense	likely pathogenic	1	inherited
	NM_000257.3	c.3346G>A	p.Glu1116Lys	missense	likely pathogenic	1	inherited
	NM_000257.4	c.2207T>C	p.Ile736Thr	missense	likely pathogenic to pathogenic	1	inherited
	NM_000257.3	c.2678C>A	p.Ala893Glu	missense	likely pathogenic	2	inherited
	NM_000257.4	c.4472C>G	p.Ser1491Cys	missense	likely pathogenic	1	inherited
	NM_000257.4	c.1207C>T	p.Arg403Trp	missense	pathogenic	1	inherited
MYBPC3 [2]	NM_000256.3	c.[821+1G>A]	N/A [7]	N/A	pathogenic	1	inherited
	NM_000256.3	c.1484G>A	p.Arg495Gln	missense	likely pathogenic to pathogenic	1	inherited
	NM_000256.3	c.1468G>A	p.Gly490Arg	missense	likely pathogenic to pathogenic	2	de novo, inherited
	NM_000256.3	c.26-2A>G	N/A	splice mutation	pathogenic	1	inherited
	NM_000256.3	c.2864_2865delCT	p.Pro955ArgfsX95	frameshift	pathogenic	1	inherited
	NM_000256.3	c.3599T>C	p.Leu1200Pro	missense	likely pathogenic to pathogenic	1	inherited
	NM_000256.3	c.3767_3769delCCA	p.Thr1256del	deletion	likely pathogenic	1	inherited
TPM1 [3]	NM_001018005.1	c.287A>G	p.Glu96Gly	N/A	likely pathogenic	1	inherited
TNNT2 [4]	NM_001001430.2	c.274C>T	p.Arg92Trp	missense	pathogenic	3	inherited
	NM_001001430.2	c.311C>T	p.Ala104Val	missense	likely pathogenic	3	inherited
	NM_001001430.2	c.291G>T	p.Lys97Asn	N/A	likely pathogenic	1	inherited
MYL2 [5]	NM_000432.2	c.173G>A	p.Arg58Gln	missense	pathogenic	1	de novo
TNNI3 [6]	NM_000363.4	c.557G>A	p.Arg186Gln	missense	pathogenic	1	inherited

NM_000363.4	c.587A>G	p.D196G	N/A	likely pathogenic	1	inherited

Detailed list of affected genes in pediatric onset hypertrophic cardiomyopathy patients, including NCBI (The National Center for Biotechnology Information) Reference Sequence Transcript, nucleotid change, their effect on protein modification and mutation type. [1], ß-myosin heavy chain; [2], myosin binding protein C; [3], cardiac troponin T2; [4], cardiac troponin I3; [5], α tropomyosin; [6], myosin light chain 2; [7], not available; [8], considering the presence of a positive molecular genetic test of the parents and / or positive family history for HCM phenotype in parents; [9], considering the presence of a negative molecular genetic test of the parents and / or negative family history for HCM phenotype in parents.

Table S2. Clinical characteristics and imaging parameter of pediatric hypertrophic cardiomyopathy patients with compound mutations.

**Patients characteristics**	**Patient 1**	**Patient 2**	**Patient 3**	**Patient 4**
Sex (Male/Female)	Male	Female	Male	Male
Age at first diagnosis (years)	6.0	7.0	1.0	0.3
Age at last follow up (years)	39.6	22.6	14.6	26.6
Genotype / phenotype expression	Genotype positive / phenotype positive	Genotype positive / phenotype positive	Genotype positive / phenotype positive	Genotype positive / phenotype positive
Identified mutation	MYH7 [21] + TNNT2 [22]	MYH7 + TNNI3 [23]	MYH7 + TNNT2	MYH7 + TNNT2
Mutation origin	Inherited [24]	Inherited	Inherited	Inherited
**Family history**				
Positive for HCM [1]	Yes	Yes	Yes	Yes
Positive for SCD [2]	Yes	No	Yes	Yes
**Mortality**				
Death	No	No	Yes	No
**Arrhythmia** [3]				
None, Mild [4], Severe [5]	Severe	Severe	None	Severe
MAEs [6]	No	Appropriate ICD discharge	SCD	Appropriate ICD discharge
**Morbidity**				
Hospitalization	Yes	Yes	No	Yes
Age at first hospitalization (years)	37.8	7.0	-	10.9
ICD [7] implantation	Yes	Yes	No	Yes
Age at ICD implantation (years)	37.8	14.3	-	10.9
Primary or Secondary prevention	Primary	Secondary	-	Secondary
Appropriate discharge	No	Yes	-	Yes
Number of cardiac medications	1	2	0	1
NYHA [8] class I, II, III, IV	II	II	I	II
**Morphology**				
CMR [9] EDVI [10] (mL/m^2)	N/A [25]	64.0	63.0	N/A
CMR ESVI [11] (mL/m^2)	N/A	16.0	16.0	N/A
LVOTO [12]	No	No	No	No
**Hypertrophy**				
CMR myocardial mass (g/m^2)	N/A	117.0	43.0	N/A
TTE [13] IVSd [14] z-score	4.1	3.3	3.1	4.9
TTE LVPWd [15] z-score	2.5	3.3	3.2	1.7
**Fibrosis**				
CMR LGE [16]	N/A	LGE	LGE	N/A
CMR LGE localization	N/A	Entire myocardium	Uncertain detection	N/A

**Systolic function**				
TTE EF[17] (%)	61.0	57.0	78.0	41.0
GLS [18] average	N/A	N/A	N/A	-8.2
GLS dispersion	-18.0	N/A	-4.0	-2.0
GLS minimum	-13.0	N/A	-17.0	-9.0
GLS maximum	5.0	N/A	-13.0	-7.0
**Diastolic function**				
TTE MV [19] E/A Ratio	2.0	N/A	1.1	2.0
TTE MV E Deceleration (m/s)	195.0	N/A	217.0	255.0
TTE E maximum (m/s)	0.8	N/A	0.6	0.5
LA [20] Diameter (cm)	4.6	N/A	4.3	5.1

[1], hypertrophic cardiomyopathy; [2], sudden cardiac death; [3], arrhythmia on Holter or cardiopulmonary exercise test; [4], premature ventricular or supraventricular beats; [5] non-sustained or sustained ventricular or supraventricular tachycardia; [6], resuscitation and/or appropriate implantable cardioverter defibrillator discharge and/or sudden cardiac death; [7], implantable cardioverter defibrillator; [8], New York Heart Association or Modified Ross classification according to age; [9], cardiac magnetic resonance imaging; [10], end-diastolic volume index; [11], end-systolic volume index; [12], left ventricular out-flow tract obstruction; [13], transthoracic echocardiography; [14], end-diastolic inter-ventricular septal; [15], end-diastolic left ventricular posterior wall thickness; [16], late gadolinium enhancement; [17], ejection fraction; [18], global longitudinal strain; [19], mitral valve; [20], left atrium; [21], β myosin heavy chain; [22], cardiac troponin T2; [23], cardiac troponin I3; [24], presence of a positive molecular genetic test of the parents and / or positive family history for HCM with clinically conspicuous parents; [25], not available.

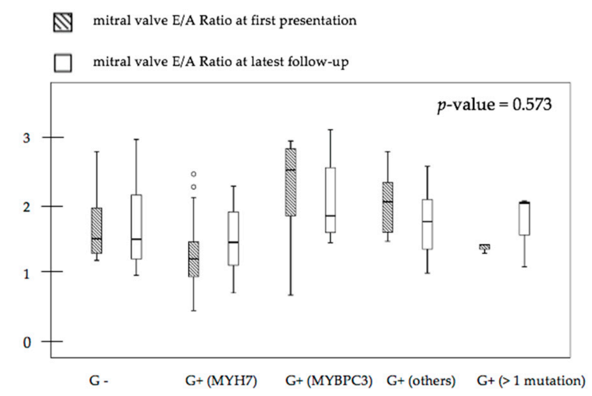

(**a**)

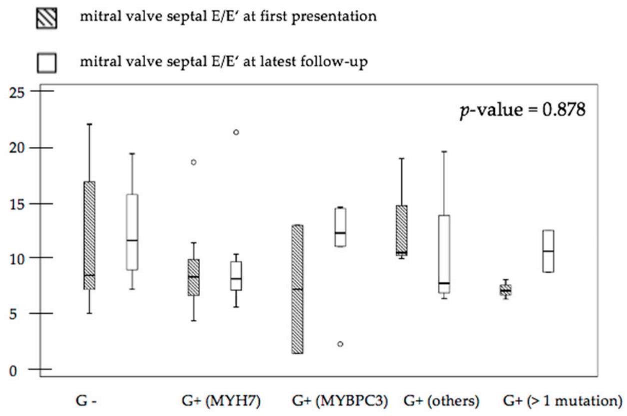

(**b**)

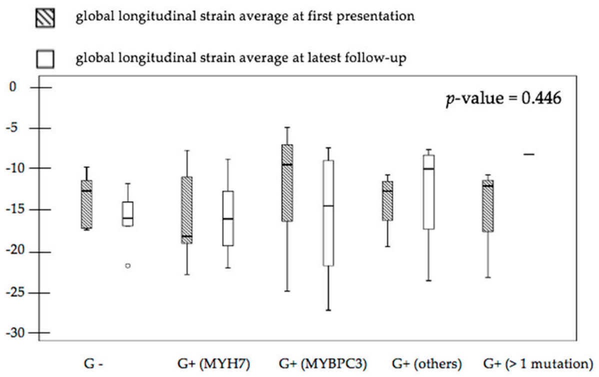

(**c**)

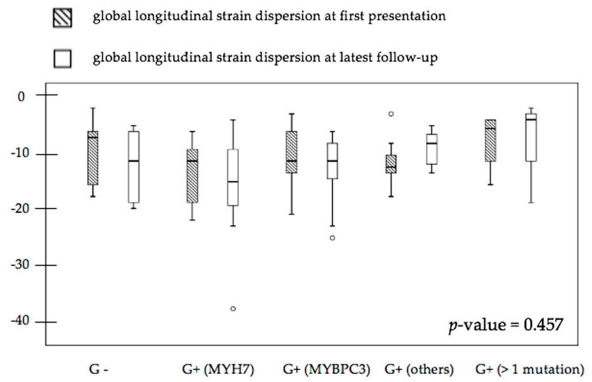

(**d**)

Figure S1. Disease progression Part II. Shown are distinct disease features at the time of first and last presentation grouped according to underlying genotype. Left ventricular diastolic function is presented by echocardiographic measurements of the mitral valve E/A ratio (**a**), and septal E/E′ ratio (**b**) on tissue Doppler imaging. Global longitudinal strain average (**c**) and global longitudinal strain dispersion (**d**) present echocardiographic signs of left ventricular systolic function. P-values were calculated with non-parametric Kruskal-Wallis test. The delta of respective parameter between first presentation and last follow up did not differ between consecutive groups. G−: genotype-negative patients, G+ MYH7: genotype-positive patients with β-myosin heavy chain single-mutation, G+ MYBPC3: genotype-positive patients with myosin binding protein C single-mutation, G+ others: genotype-positive patients with cardiac troponin T2, cardiac troponin I3, α tropomyosin and myosin light chain 2 single-mutations, G+ multiple mutation: genotype-positive patients with compound mutations, °: outliers.

Arrhythmia

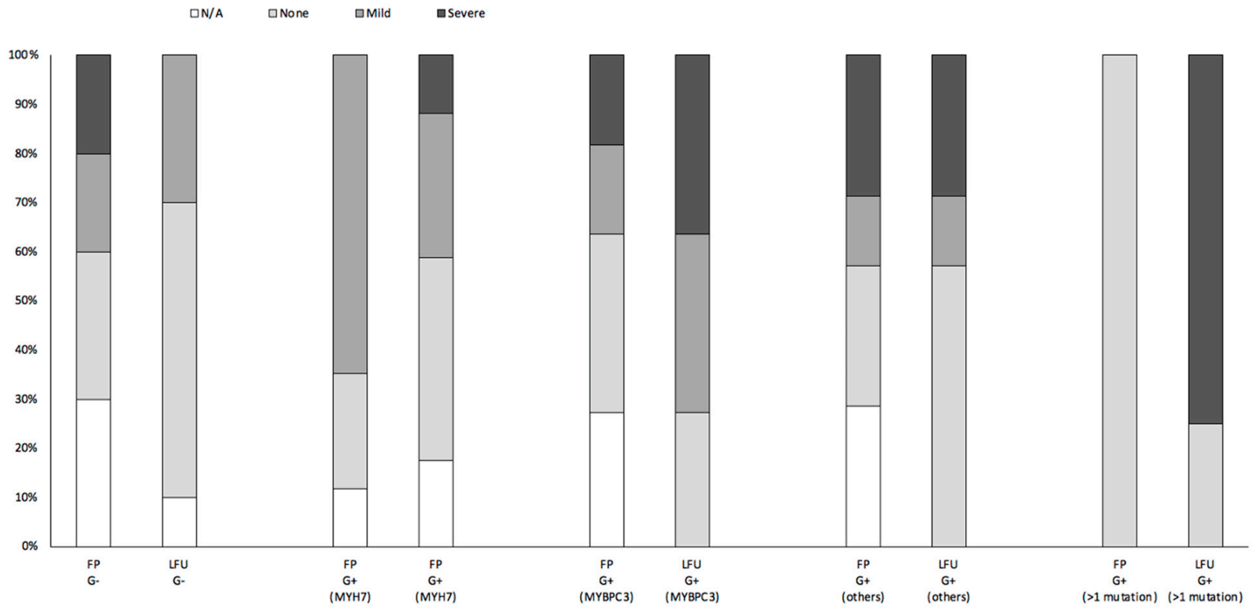

Figure S2. Progress of arrhythmia in patients with pediatric onset hypertrophic cardiomyopathy. Shown are defined arrhythmia classes on first and last presentation grouped according to underlying genotype. Arrhythmia were detected on Holter or cardiopulmonary exercise test. N/A, not available; mild, premature ventricular or supraventricular beats; severe, non-sustained or sustained ventricular or supraventricular tachycardia. P-values were calculated with non-parametric Kruskal-Wallis test. The delta of respective parameter between first presentation and last follow up did not differ between consecutive groups (*p*-value 0.179). G−: genotype-negative patients, G+ MYH7: genotype-positive patients with β-myosin heavy chain single-mutation, G+ MYBPC3: genotype-positive patients with myosin binding protein C single-mutation, G+ others: genotype-positive patients with cardiac troponin T2, cardiac troponin I3, α tropomyosin and myosin light chain 2 single-mutations, G+ multiple mutation: genotype-positive patients with compound mutations.

References

1. Richards S, Aziz N, Bale S, Bick D, Das S, Gastier-Foster J, Grody WW, Hegde M, Lyon E, Spector E *et al*: Standards and guidelines for the interpretation of sequence variants: a joint consensus recommendation of the American College of Medical Genetics and Genomics and the Association for Molecular Pathology. *Genet Med* **2015**, *17*(5):405-424.
2. Amendola LM, Jarvik GP, Leo MC, McLaughlin HM, Akkari Y, Amaral MD, Berg JS, Biswas S, Bowling KM, Conlin LK *et al*: Performance of ACMG-AMP Variant-Interpretation Guidelines among Nine Laboratories in the Clinical Sequencing Exploratory Research Consortium. *Am J Hum Genet* **2016**, *99*(1):247.
